# Slow-Release Nitrogen Fertilizers with Biodegradable Poly(3-hydroxybutyrate) Coating: Their Effect on the Growth of Maize and the Dynamics of N Release in Soil

**DOI:** 10.3390/polym14204323

**Published:** 2022-10-14

**Authors:** Soňa Kontárová, Radek Přikryl, Petr Škarpa, Tomáš Kriška, Jiří Antošovský, Zuzana Gregušková, Silvestr Figalla, Vojtěch Jašek, Marek Sedlmajer, Přemysl Menčík, Mária Mikolajová

**Affiliations:** 1 Institute of Materials Chemistry, Faculty of Chemistry, Brno University of Technology, 61200 Brno, Czech Republic; 2 Department of Agrochemistry, Soil Science, Microbiology and Plant Nutrition, Mendel University in Brno, 61200 Brno, Czech Republic; 3 Institute of Natural and Synthetic Polymers, Faculty of Chemical and Food Technology, Slovak University of Technology in Bratislava, Radlinského 9, 812 37 Bratislava, Slovakia

**Keywords:** slow-release, nitrogen fertilizers, coating, poly(3-hydroxybutyrate), ammonium nitrate, biodegradable, dioxolane, biomass, control-release, maize

## Abstract

Fertilizers play an essential role in agriculture due to the rising food demand. However, high input fertilizer concentration and the non-controlled leaching of nutrients cause an unwanted increase in reactive, unassimilated nitrogen and induce environmental pollution. This paper investigates the preparation and properties of slow-release fertilizer with fully biodegradable poly(3-hydroxybutyrate) coating that releases nitrogen gradually and is not a pollutant for soil. Nitrogen fertilizer (calcium ammonium nitrate) was pelletized with selected filler materials (poly(3-hydroxybutyrate), struvite, dried biomass). Pellets were coated with a solution of poly(3-hydroxybutyrate) in dioxolane that formed a high-quality and thin polymer coating. Coated pellets were tested in aqueous and soil environments. Some coated pellets showed excellent resistance even after 76 days in water, where only 20% of the ammonium nitrate was released. Pot experiments in Mitscherlich vegetation vessels monitored the effect of the application of coated fertilizers on the development and growth of maize and the dynamics of N release in the soil. We found that the use of our coated fertilizers in maize nutrition is a suitable way to supply nutrients to plants concerning their needs and that the poly(3-hydroxybutyrate) that was used for the coating does not adversely affect the growth of maize plants.

## 1. Introduction

The importance of fertilizers for the human population is indisputable. Without the Haber–Bosch synthesis of ammonia (commercialized in 1913), and the subsequent synthetic nitrogen compounds and their applications, we would not be able to produce more than half of today’s world food [1]. Due to the rapid growth of the population by 2–3 million people in the next 80 years, more crop production will be needed, which will also increase the need for fertilizers [2,3]. Fertilizers provide plants with fundamental macronutrients (nitrogen/phosphorus/potassium), where nitrogen is essential to plant growth [4]. However, crops’ poor absorption of nutrients from fertilizers and the related losses of nitrogen in the soil, water, and air caused a cascade of environmental problems [5]. Despite the fact that the use of nitrogen fertilizers plays an essential role in meeting the demand for crop production, nitrogen use efficiency is relatively low due to their excessive use (in general between 25 and 50%), which often leads to losses of redundant nitrogen from agroecosystems [6]. Environmental problems include eutrophication and the expansion of dead zones in coastal ocean waters resulting from the leaching of nitrates into rivers, lakes, ponds, and ground waters. The atmospheric deposition of ammonia and nitrates affects natural ecosystems; nitrous oxide (N_2_O) is now the third most crucial greenhouse gas, following CO_2_ and CH_4_ [1,4]. The excessive use of fertilizers can cause soil acidification, depletion of soil cations, reduction of carbon uptake, and contamination with heavy metals. All this negatively influences animal and human health, the environment, biodiversity, and the climate [7,8]. Even more significant losses of nitrogen in the environment (that will occur with a growing population) could be reduced by more moderate meat consumption, low-protein animal feeding, better agronomic management, and a portfolio that includes split applications and balanced use of fertilizers, precision farming, optimized crop rotations, and the use of expensive slow-release compounds [1].

Controlled- and slow-release fertilizers (CRFs and SRFs) provide a more efficient, economical, and safe way to deliver nutrients to plants. They are able to retain nutrients in the soil for a longer period, as they are available for the plants at the desired rate or concentration level. Thus, nutrient utilization efficiency (NUE) improves due to less frequent dosing and reduced nutrient removal from the soil by rain or irrigation, which also reduces environmental hazards [4,9,10,11,12]. Slow- or controlled-release fertilizers are a good alternative to conventional mineral fertilizers, and their use allows for a reduction of the fertilizer rate by 20% or 30% of the recommended value to achieve the same yield [13]. Slow-release fertilizers are described as “low solubility compounds with a complex/high molecular weight chemical structure that releases nutrients through either microbial or chemically decomposable compound” [4,14]. Controlled-release fertilizers (CRFs) can be described as “products containing sources of water-soluble nutrients, the release of which in the soil is controlled by a coating applied to the fertilizer” [4,15]. SRFs are usually classified into “condensation products of urea-aldehydes, fertilizers with a physical barrier (coated or incorporated into the matrix), and super granules. CRF is a subset of SRF, which falls under the category of fertilizer with a physical barrier” [4].

Current commercial CRFs with polymer coatings are often made of a thermoplastic resin such as polyolefin, polyvinylidene chloride, and copolymers, which cannot degrade easily in soil and accumulate over time. Generally, these CRFs containing synthetic, non-biodegradable polymers accumulate up to 50 kg/ha per year in the soil after releasing their nutrients, remaining and causing white pollution [4,16]. With the agreement of the European Green Deal, EU Fertilizing Products Regulation (FPR), and Circular Economy Action Plan, manufacturers are faced with significant challenges; they will have to adapt their current practices to the new FPR requirements, including biodegradability criteria for polymer coatings of controlled-release fertilizers [4,17].

The present study aimed to prepare CRFs with a coating of a biodegradable nature. The pelletization of powder fertilizer ammonium nitrate with dolomite (CAN) with selected additive materials (poly(3-hydroxybutyrate) P3HB, struvite, dried biomass containing P3HB) has become the most technologically feasible. Using struvite and biomass from P3HB extraction meets the requirements of the circular economy. P3HB can be made from waste cooking oil and a variety of byproducts [18,19,20,21]. However, pellets alone without a non-defective coating would not meet the requirements of slow release. A 6% solution of fully biodegradable P3HB with green solvent dioxolane [22] was applied to the pellets by 6-fold immersion [23] and produced a good quality coating.

The kinetics of fertilizer release from coated pellets in an aqueous environment was monitored by the conductometry method. The results showed the high quality of the prepared water-barrier polymer coating. The dynamics of mineral nitrogen release of these CRFs were studied under laboratory conditions, followed by an evaluation of the effectiveness of the newly developed coated fertilizers on maize growth and an assessment of their effects on soil mineral nitrogen content. We assumed that the coated CAN-based fertilizers would reduce the environmental load in the soil without simultaneously limiting plant growth.

## 2. Materials and Methods

### 2.1. Preparation of Coated and Encapsulated Fertilizers

#### 2.1.1. The Fertilizer

The fertilizer used in pellets was calcium ammonium nitrate (CAN) supplied by Lovochemie, a.s. (Lovosice, Czech Republic) [24]. CAN is a nitrogen fertilizer containing 27% of nitrogen (up to 13.5% N-NH_4_^+^ and 13.5% N-NO_3_^−^) and 21% of dolomite (CAS 16389-88-1). It is a mixture of ammonium nitrate with finely ground dolomite in the form of whitish to light brown granules. Most particles (up to 90%) of CAN had a size in the range of 2–5 mm. Before pelletization, it was necessary to grind CAN into a powder on a grain mill (Sana Products Ltd., České Budějovice, Czech Republic) and sieve (Sana mesh sieve, pore size 0.5 × 0.5 (mm × mm)). Thus, the CAN was finally below 0.5 mm.

#### 2.1.2. Filling Material in Pellets

The primary filler material (together with the fertilizer) in the pellets prepared manually using a hydraulic press was poly-3-hydroxybutyrate (P3HB, *ρ* = 1.23 g∙cm^−3^, M_w_ = 450,000 g·mol^−1^, purity 98–99%) supplied by TianAn Biopolymer (Ningbo, People’s republic of China) [25] and commercially purified in acetone as a white powder (Nafigate Corporation, Prague, Czech Republic) [26].

Another filler material used in some fertilizer mixtures was a struvite (NH_4_MgPO_4_ · 6H_2_O). A homogenized mixture of struvite from Nafigate and Aldrich (Merck KGaA, St. Louis, Missouri, USA) [27], in the form of a white powder, free of lumps by sieving, was used.

Dried biomass was also used in some mixtures with fertilizer as a filler material. The biomass was supplied by Nafigate Corporation and was produced from waste cooking oil by fermentation with a bacterial culture of *Cupriavidus necator* H16. The specific procedure for biomass production is described in [18]. After cultivation, the biomass was concentrated by centrifugation to 50% dry matter and stored frozen at −20 °C. It was dried at 105 °C to constant weight before use. Before pelletization, the lumps were removed by sieving. The dried biomass contained about 60% P3HB.

#### 2.1.3. Preparation of Fertilizer Pellets

Pelletization was performed by compressing powdered materials of fertilizer CAN and other filler materials (P3HB, struvite, dried biomass containing P3HB) into pellets using a manual hydraulic press SPECAC (Orpington, Great Britain) [28] with a compressive force equivalent to 2 tons. Each resulting pellet had weight = 0.5 g, height = 4 mm, and diameter = 10 mm (approx.). Before the powder mixture preparation and homogenization, the fertilizer powder and P3HB powder were dried in an oven at 60 °C overnight and freed of lumps with a sieve (other filler materials: struvite and biomass were also sieved, and the sieve pore size was 0.5 × 0.5 (mm × mm)). Several series of cold-pressed pellets were prepared and composed of: 50% fertilizer CAN + 50% P3HB, 50% CAN + 50% biomass, 50% CAN + 25% P3HB + 25% struvite and 100% CAN serving as reference.

#### 2.1.4. The Material for the Coating Solution

Coatings were prepared with a 6% solution of P3HB dissolved in chloroform with amylene ((CHCl_3_, M_r_ = 119.38; 99.93%, stabilized with amylene max. 55 ppm, supplied by Lach-Ner Ltd. (Neratovice, Czech Republic) [29]), then a 7% solution of P3HB in chloroform with ethanol ((99.8%, stabilized with approx. 1% ethanol p.a., supplied by PENTA Ltd. (Chrudim, Czech Republic) [30]) and 6% solution of P3HB in 1,3-dioxolane ((C_3_H_6_O_2_, M_w_ = 74.08 g·mol^−1^, density 1.07 kg/L, stabilized with 0.03% BHT (2,6-Di-Tert-Butyl-4-Methylphenol), supplied by VWR International Ltd. (Stříbrná Skalice, Czech Republic) [31]). The solutions were prepared for preliminary tests of coating solutions and for dip coating of pelletized fertilizers purposes.

#### 2.1.5. Coating of Fertilizer Pellets and Encapsulation

Finally, after preliminary tests of coating solutions, dioxolane was chosen as the best solvent for preparing the coating solution. The prepared pellets were coated manually by dipping them six times in a beaker with a 6% coating solution of P3HB in dioxolane.

The preparation of 6% P3HB in dioxolane for manual coating was first carried out by dissolving in an oven at a temperature of 90–95 °C approx. 1.5–2 h. However, new and faster ways of preparing larger volumes of solutions (at least 2000 mL) for later coating in a coating drum were sought (will be part of another article).

The resulting coating solution had suitable viscosity, and the final coating weight was only 12–17% of the original pellet weight (depending on the composition of the pellet to which the coating was applied).

The prepared pellets of 50% CAN + 50% P3HB were also encapsulated in an experimental biodegradable film based on P3HB, thermoplastic starch TPS, and PLA instead of coating. The biodegradable film was prepared by Panara Nitra (Slovakia, [32]) with Slovak University of Technology in Bratislava (Bratislava, Slovakia, [33]). The intention was to use biodegradable films based on P3HB and auxiliary polymers that were developed for complete decomposition in environmental components. These foils were prepared to use fully degradable agricultural foils, both in compost and in soils. An annular welding head was tailor-made for encapsulation that was electrically heated to a suitable temperature close to the melting point of the film (approx. 160 °C). By applying the appropriate pressure and time, a defect-free connection around the entire pellet was achieved. These encapsulated pellets were also tested in aqueous and soil environments.

### 2.2. Testing Nitrogen Release from Coated Fertilizer

The conductometric method monitored the release of ammonium and nitrate nitrogen in the aqueous medium from our six times hand-coated and encapsulated pellets prepared on a hydraulic press.

Ammonium nitrate is an inorganic, highly soluble fertilizer that dissociates into individual ions in aqueous environments: ammonium (NH_4_^+^) and nitrate (NO_3_^−^). The solubility of ammonium nitrate is 213 g/100 g H_2_O [34], and conductometry can be used to determine the solvated fraction. Conductometry is a method based on measuring the conductivity of the second type of conductor: a solution whose conductivity is ensured by the ions present. The specific conductivity of the solution (*κ*) is related to the conductance (*G*), which can be determined by measuring the electric current flowing between the two electrodes of the conductometric probe through free charge carriers (ions). The conductance (*G*) is a quantity depending on the geometry of the measuring probe, and therefore it is used only to obtain a specific conductance (*κ*). Since, in the case of fertilizer analysis, there is a fundamental relationship between ion concentration (*c*_*Ion*_) and specific conductance (*κ*), we describe the dependence of conductivity on ion concentration by the molar conductivity quantity (Λ*_m_*):

(1) $$G=\kappa \cdot \frac{A}{l}$$

*G*…conductance (S)
*κ*…specific conductivity (S · cm^–1^)
*A*…probe electrode area (cm^2^)
*l*…probe electrode distance (cm)

(2) $$\kappa =\Lambda_{m}\cdot c_{Ion}$$

Λ*_m_*…molar conductivity (S · cm^2^ · mol^−1^)
*c_Ion_*…ion concentration (mol · cm^−3^) [35]

The measurements of nutrient loss from the prepared pellets were performed in closable plastic test tubes with a volume of 50 mL. For all tests, one pellet (approx. 0.5 g) of fertilizer compressed into the form of a carrier with additives was placed in test tubes with a volume of distilled water of 40 mL. Ten pellets were measured for each experimental formulation, and the results were averaged. The measurements were performed at laboratory conditions (temperature 22 °C). The current signal was electronically converted to a frequency. The dependence of the change in the frequency of the alternating current on time was measured using a METEX^®^ M-3850D multimeter (Metex Corporation, Toronto, Canada) [36]. Values were recorded initially after 24 h, and then the frequency of monitoring was reduced as necessary, and the time interval between measurements was usually 48 h. The method of calibration dependence between the frequency of alternating current and electrolyte concentration was used to monitor the mass transport of the nutrients from controlled release fertilizer. The calibration dependences were fitted using the Origin program (OriginLab, Northampton, MA, USA) [37] with two exponential fits to obtain the calibration dependence equation. Finally, the weight loss of the fertilizer was determined from this calibration dependence equation. We counted 21% dolomite content in the CAN fertilizer in the calculations of ammonium and nitrate loss from the pellets.

### 2.3. The Greenhouse Pot Experiment

The pot greenhouse experiment aimed to verify the effect of the application of coated fertilizers on the development and growth of maize and the dynamics of N release in the soil. A pot experiment was established in the vegetation hall of Mendel University in Brno (Brno, Czech Republic), evaluating the effect of fertilizing of coated fertilizers applied during the sowing period of maize.

#### 2.3.1. Experimental Design

**Figure 1:**
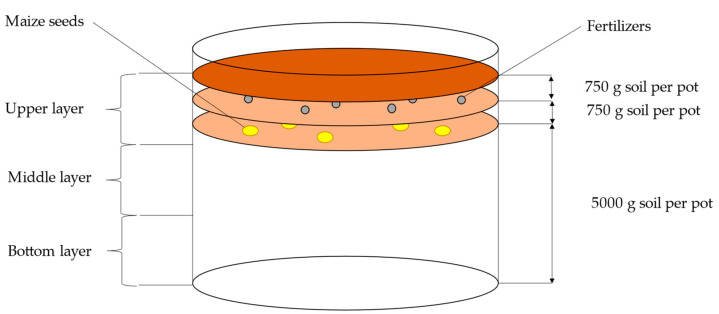
Scheme of Mitscherlich vegetation pot.

The experiment was performed in Mitscherlich vegetation pots (on 6.5 kg of soil). Five kg of soil was weighed into each pot. Maize (6 seeds per pot) was sown on the soil surface followed by uniform coverage with soil (750 g). Fertilizers were manually spread on the surface of this layer and evenly covered with another 750 g of soil (Figure 1). The basic agrochemical properties of the soil used in the pot experiment are presented in Table 1. SY Orpheus maize variety from Syngenta (Oseva, a.s., Bzenec, Czech Republic) [38] was used in the experiment. The pot experiment was performed under semi-natural conditions (rain shelter) in the vegetation hall. An identical controlled watering regime was used for all pots during the experiment. Plants were watered to 70% of maximum water holding capacity throughout the growing season. The pots were hand-watered with demineralized water on the soil surface.

The fertilization treatments used in the experiment are presented in Table 2.

Each of these treatments was based on 12 replicates (pots) distributed in the vegetation hall at random. Maize was sown on 18 March 2021. The influence was monitored of the applied fertilizers on plant growth and nitrogen content in the soil profile after the establishment of the experiment (sowing) in regular 3-week intervals (t_1_ = 8 April; t_2_ = 29 April; and t_3_ = 20 May 2021). After emergence (29 March 2021), the maize plants were adjusted to a final number of three plants per pot. Soil and plant analyses were performed during the vegetation.

#### 2.3.2. Soil and Plant Sampling and Analyses

Soil sampling was carried out at the observed intervals (t_1_–t_3_) for each variant (each variant from four pots in each term). Soil samples were collected from each pot from three soil profile depths (upper, middle, and bottom layer, Figure 1). The contents of mineral nitrogen (Nmin)—ammonium (NH_4_^+^) and nitrate (NO_3_^−^) were determined in the soil samples [42].

The effects of the coated fertilizers on the growth of maize plants were monitored during the experiment at identical intervals (terms t_1_–t_3_). Chlorophyll content (N tester value), vegetative index (NDVI), basic photosynthetic parameters based on the action and measurement of light signal in PS II, dry weight of maize aboveground biomass (AGB), nitrogen content in AGB and capacitance of the root system were evaluated in plants (Table 3).

#### 2.3.3. Statistical Data Analysis

The soils and plants were analyzed in STATISTICA 12 software [46] using Tukey’s analysis of variance followed by testing at the 95% level of significance (*p* ≤ 0.05). Normality and homogeneity of variances were checked using the Shapiro–Wilk test and Levene’s test. In the case of the greenhouse pot experiment, the results are expressed as arithmetic mean ± standard error (SE).

## 3. Results

Several mixtures (formulations) of fertilizer and filler materials were successfully prepared by compressing powdered fertilizer (CAN) with filler materials (P3HB, struvite, dried biomass containing P3HB) into pellets using a manual hydraulic press. The coating was performed manually by dipping them six times in a beaker with a 6% coating solution of P3HB in dioxolane.

### 3.1. Preliminary Tests of Coating Solutions

Dioxolane was finally chosen as a solvent for P3HB in the coating solution based on preliminary tests that compared coating solutions of 6% P3HB in dioxolane, 7% P3HB in chloroform with ethanol, and 6% P3HB in chloroform with amylene. A solution of 6% P3HB in dioxolane was prepared for this test in an oven at 90–95 °C for 2 h (however, now, we are able to reduce the time required to prepare the solution to only 25 min using stirred autoclave with a heating jacket). Coating solutions of P3HB with chloroform were prepared under reverse reflux at 65 °C for 1 h. Pellets prepared on a manual press composed of 50% CAN + 50% P3HB were selected for preliminary coating solution tests. After preparing the individual P3HB coating solutions, the pre-weighed pellets were soaked in beakers with these individual solutions, pulled out after 2–5 s, and dried on glass material for at least 30 min (under laboratory conditions). After each soaking and drying (coating layer formation), the pellets were weighed again. Six layers of the coating were applied in this way. The pellets were weighed again the next day to determine the final weight of the pellets after applying six coats of coating to ensure all solvents were evaporated. For clarity, we present the final evaluation of coating weights of the pellets after applying six layers of coating (see Table 4) and the overall evaluation of individual coating solutions.

A solution of 7% P3HB in chloroform with ethanol seemed to be more gel-like. It was harder to apply and evaporated more slowly but created a quality coating that did not crack. Still, the resulting coating is too heavy. Boyandin et al. [23] acquired after the application of 6 layers approx. 60% extra pellet weight. A solution of 6% P3HB in dioxolane was applied well and quickly (lower viscosity), evaporated faster than chloroform, created a quality coating that does not crack, and the significant advantage was that the resulting 6-times coating was only (19.9 ± 1.5)% of the weight of the original pellet. A solution of 6% P3HB in chloroform with amylene was unusable; the solution was applied well at first, but the coating cracked at the second layer and was highly defective (see Figure S1). Although six-layer pellets were finally weighed, the resulting weight was irrelevant.

Although these individual solutions were not tested on a large number of pellets, the differences were so significant that in terms of quality and weight of the formed 6-times coating, a solution of 6% P3HB in dioxolane was a clear choice. A solution of 7% P3HB in chloroform with ethanol also produced a quality coating. However, due to the enormous weight of the applied coating (due to the high viscosity of the solution), this solution is disadvantageous and not so well used for manual coating on a laboratory scale, even when coating in a coating drum. Using a solution of P3HB in dioxolane, which has a suitable viscosity, we achieved a high-quality multiple coating: only 20% of the extra coating weight on the pellet is a significant improvement. Therefore, the solution of P3HB in dioxolane was best for application; dioxolane evaporated the fastest, and the resulting 6-times coating had excellent quality and the lowest weight. Legislative changes (Green Deal) have also contributed to the choice of dioxolane as a coating solvent. The advantage is also lower health risks when working with dioxolane. The disadvantages are its flammability and high price compared with chloroform.

### 3.2. Slow-Release Fertilizers

**Figure 2:**
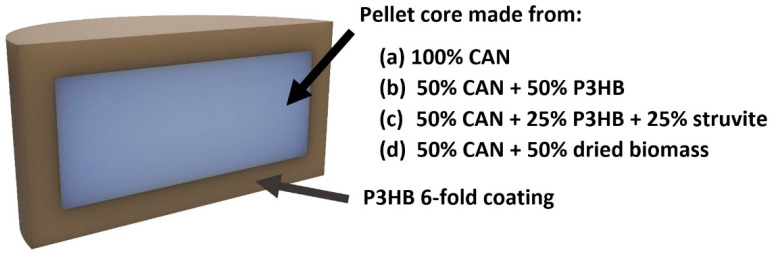
Schematic representation of coated pellets.

Pellets of various formulations were successfully prepared on a hydraulic press for further testing in aqueous and soil environments. These pellets contained CAN fertilizer, P3HB, struvite, and biomass in different composition. Approximately 120 pellets for each formulation were prepared. The pellets were coated manually with six layers using a solution of P3HB in dioxolane. Schematic representation of coated pellets can be seen in Figure 2 and coated pellets of different experimental formulations in Figure S2. Details of the pellet preparation and coating process were already described in the experimental part.

From each formulation, ten pellets were weighed before manual coating, and after pellets were coated with six layers of P3HB coating in dioxolane. The weighing of the coated pellets did not take place until the second day after manual coating so that the solvent could evaporate. Table 5 presents the calculated arithmetic mean ± standard deviation of the weight of the 6-fold coating applied to the pellets of different formulations (% of the original weight of the pellets). The resulting 6-fold coating of P3HB in dioxolane had finally even less weight than expected. In the case of pellets 50% P3HB with 50% CAN, the coating was approx. 17% by weight of the original pellet. For pellets that contained 50% CAN + 25% P3HB + 25% struvite or 50% CAN + 50% biomass, the resulting coating had approx. 14% by weight of the original pellet. Finally, for pellets containing 100% CAN fertilizer, the coating had only approximately 12% by weight of the original pellet.

### 3.3. Pellets Encapsulated in Foils

A special foil was used for the manual encapsulation of 0.5 g pellets with a composition of 50% CAN + 50% P3HB instead of coating (details described in Section 2). Pellets were created on a hydraulic press in the laboratory, and an experimental biodegradable film marked NR/75/267 (Manufacturer Panara Nitra, Slovakia, P3HB content 13%, thermoplastic starch TPS 22%) was used for encapsulation. The film achieves 50% decomposition in the soil, measured on the basis of exhaled CO_2_ after 420 days. The biodegradability test leading to the complete decomposition of the film has not yet been completed.

The first versions of the pellets with the starch-containing films were too permeable in the weld. Foil welds were first solved by folding the foil and closing with a weld on three sides. As it turned out, it was not possible to prepare a weld without a defect in the corners. Therefore, the encapsulation geometry was changed to a circular shape. Thus, an annular welding head was made to measure, which was electrically heated to a suitable temperature close to the melting point of the film (approx. 160 °C). Both welded foils were placed in the mating piece between the Teflon foils to prevent the molten foil from adhering to the welding head and the mating piece. A defect-free connection around the entire pellet was achieved by applying the appropriate pressure and time. Thus, 120 pellets with a composition of 50% fertilizer CAN + 50% P3HB were encapsulated in the film for further testing in aqueous and soil environments (see Figures S3 and S4).

### 3.4. The Nitrogen Release from Coated Fertilizer in the Aquatic Environment

Measuring the loss of fertilizer from the prepared defined carriers in the aquatic environment (distilled water, laboratory conditions, conductometry method described in the experimental part) provided us only an indicative prediction for the subsequent use of fertilizers in the soil in terms of their “slow-release” quality. Nevertheless, it could early on be deduced from these tests whether the pellets and their subsequent coating were designed correctly in terms of their chemical composition and whether they were well prepared and coated so that there would be a gradual release of nitrogen in the soil.

**Figure 3:**
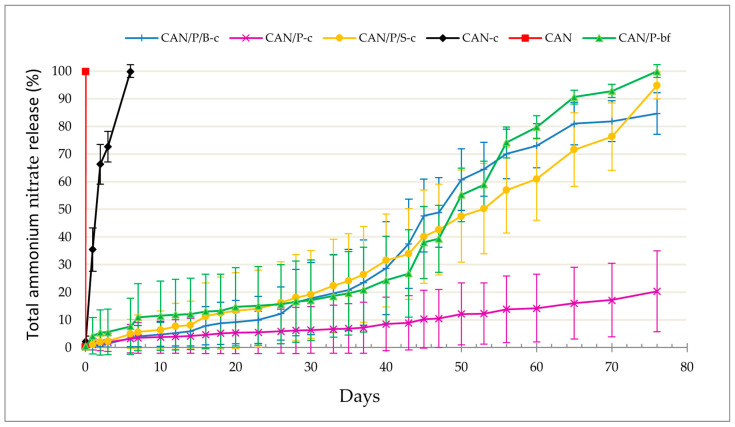
Release of ammonium nitrate (AN) from pellets of different compositions in the aquatic environment as a function of time. Total ammonium nitrate release in% is expressed as mean (n = 10), and the error bars represent the standard deviation. CAN-c: 100% CAN with P3HB coating; CAN/P-c: 50% CAN + 50% P3HB with P3HB coating; CAN/P/S-c: 50% CAN + 25% P3HB + 25% struvite with P3HB coating; CAN/P/B-c: 50% CAN + 50% biomass with P3HB coating; CAN/P-bf: 50% CAN + 50% P3HB encapsulated in biodegradable film; CAN: 100% CAN (positive reference).

The compositions of the individual manually coated fertilizer formulations prepared on a hydraulic press that were tested in the aqueous medium can be seen in Table 6. The release of ammonium nitrate (AN) in the aqueous medium from our six-fold manually-coated pellets (containing CAN fertilizer) was investigated by the conductometric method. Figure 3 shows the results of the measurement completed on day 76.

As can be seen, all ammonium nitrate fertilizer was released immediately (in 1 h in the aquatic environment) from the reference (100% CAN without coating). The rapid release of fertilizer was also observed in coated pellets containing 100% CAN. Half of the AN was released from these pellets after approximately 1.5 days and all in 6 days. The adhesion of the 6-fold coating prepared on these pellets containing 100% CAN fertilizer was probably not as great as for pellets containing P3HB in addition to the fertilizer.

The release of AN from the coated pellets containing struvite, biomass, and pellets with foil instead of coating showed a similar course of fertilizer release. Half of the AN was released from pellets containing struvite on day 53, from pellets containing biomass approximately day 48, and from pellets with foil approximately day 49. However, at the end of the measurement (day 76), all ammonium nitrate was released from the foil pellets, while 95% of AN was released from the struvite-containing pellets and 85% of the AN from the pellets containing biomass. Significantly, the least AN was released from coated pellets containing 50% CAN and 50% P3HB. After 76 days, only 20% of the ammonium nitrate was released. The nitrogen ammonium release curve of the fertilizer has an almost linear course in this interval (lag phase and linear phase [4]), which indicates a perfectly flawless coating. This flawless coating could have been formed due to the high adhesion of the P3HB coating (dissolved in dioxolane) to the pellets of this composition. This indicates that P3HB inside the pellet is essential as an adhesion promotor. In this case, a well-made coating acts as a polymer layer almost impermeable to water.

The results of water tests show that these pellets have the potential for the gradual release of nitrogen even in the soil environment. It is also clear that for a quality coating that adhered well to our pellets, it was necessary to add P3HB as an additive. On the 100% CAN pellets without P3HB, no coating was formed that would withstand the action of water for a long time. In comparison, the coating on the pellets containing 50% P3HB showed high resistance even after 76 days in water.

### 3.5. Efect of Coated Fertilizers in Greenhouse Experiment

Based on the identified potential of the coated and encapsulated fertilizers under aqueous test conditions, the pot vegetation experiment aimed to verify the effect of our different fertilizer formulations on the development and growth of maize and the dynamics of N release in the soil. The experiment took place in Mitscherlich’s vegetation pots in the vegetation hall at the Mendel University Brno (Figure S5).

#### 3.5.1. The Nitrogen Release from the Fertilizers in the Soil

During plant growth, soil sampling was performed at regular 3-week intervals to determine the mineral nitrogen content (described in the experimental part). Based on the content of mineral N (NH_4_^+^ and NO_3_^−^) determined in individual soil layers of the pot (upper, middle, and bottom layer) over time, the effect of coating on the dynamics of nitrogen release from our tested fertilizers was assessed.

In the first term (t_1_) of soil collection (3 weeks after sowing/fertilization), it is evident that from uncoated fertilizer (CAN), nitrogen is released very quickly and leaches in the lower layer of soil profile due to watering. The NH_4_^+^ nitrogen supply in this treatment is relatively low, which is a consequence of the rapid release of this form N from fertilizer and its rapid nitrification (Figure S6a,b).

Coated fertilizers (CAN-c, CAN/P-c, CAN/P/S-c, and CAN/P/B-c) showed the ability to gradually release N. This was evidenced by the significantly high contents of ammonium N in the upper layer of the soil. In contrast with uncoated fertilizer (CAN), NH_4_^+^ was later released from modified coated fertilizers. The gradual release of N from these fertilizers was also evidenced by the low content of nitrates in the lower soil layer (Figure S6b). Its lowest values were reached in treatment with encapsulated fertilizer CAN/P-bf. The hydrolysis of encapsulated fertilizer was slowed down, and thus, the nitrification of the released NH_4_^+^ did not occur so intensively). Thus, the CAN/P-bf fertilizer (encapsulated pellets) showed the slowest N release. This is evidenced by the content of ammonium N in the soil (all layers), which was statistically the lowest in this treatment option.

The gradual nutrient release from coated fertilizers is evident in the soil analysis results performed 6 weeks after sowing of maize (t_2_). The significantly highest NH_4_^+^ content in the upper and middle soil layer was recorded for all treatments with coated (CAN-c, CAN/P-c, CAN/P/S-c, and CAN/P/B-c) and encapsulated fertilizer CAN/P-bf (Figure S7a). There was a quite change in the content of soil N, especially at CAN-c (100% CAN with P3HB coating). The determined NH_4_^+^ content was the lowest of the coated fertilizers. This indicates its nitrification and horizontal shift (between t_1_ and t_2_). In the treatment fertilized with uncoated fertilizer (CAN), the nitrogen released from the fertilizer has long been uptake by plants or nitrified and leached into the lower layers, as evidenced by the ammonium ion supply in the soil, which was at the level of the non-fertilized treatment (CON-nf).

The highest nitrate nitrogen content in the soil was also recorded in the upper and middle part of the soil profile in treatments fertilized with coated fertilizers, especially in treatments CAN/P/S-c, and CAN/P/B-c (Figure S7b). Its increased amount in the soil is due to the gradual release of nitrates from coated CAN and gradual nitrification of ammonium N. The content of nitrates in the lower soil layer was compared between the treatments. If we compare this amount of NO_3_^−^ with the values found in the term t_1_ for treatment CAN (79.6 mg/kg), the use of coated fertilizers significantly reduced the risk of its leaching.

In soil analyses performed 9 weeks after sowing (t_3_), it is evident that the plants depleted mineral nitrogen from the soil. This is evidenced by the very low contents of the ammonium and nitrate forms of N in all soil layers (Figure S8a,b). However, for both forms of acceptable nitrogen, there are apparent differences between the fertilizers used at this time. The significantly highest supply of NH_4_^+^ and NO_3_^−^ was found on treatments fertilized with coated fertilizers (especially CAN/P/S-c, CAN/P/B-c) and encapsulated fertilizer CAN/P-bf.

**Figure 4:**
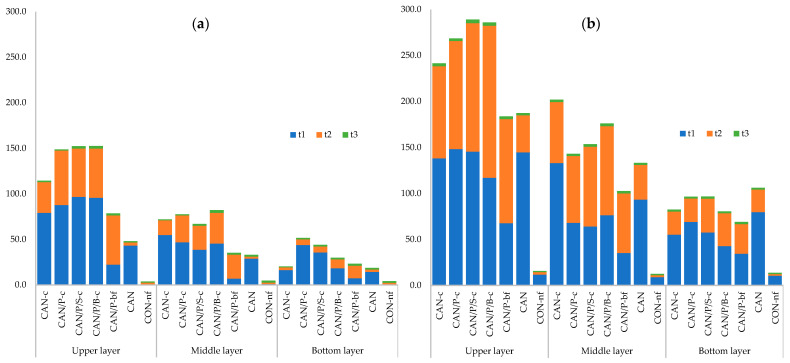
Development of ammonium (**a**) and nitrate (**b**) soil nitrogen content (mg/kg of soil) over time. CAN-c: 100% CAN with P3HB coating; CAN/P-c: 50% CAN + 50% P3HB with P3HB coating; CAN/P/S-c: 50% CAN + 25% P3HB + 25% struvite with P3HB coating; CAN/P/B-c: 50% CAN + 50% biomass with P3HB coating; CAN/P-bf: 50% CAN + 50% P3HB encapsulated in biodegradable film; CAN: 100% CAN (positive reference); CON-nf: without fertilizer (negative reference).

The dynamics of the release of the monitored forms of mineral nitrogen from fertilizers are presented in Figure 4a,b. It is clear that coated fertilizers tend to retain nitrogen in the soil (soil profile), delay its conversion into leachable N (nitrate), contribute to eliminating its loss by leaching, and thus increase the efficiency of fertilization using nitrogen by plants.

#### 3.5.2. Effect of Coated and Encapsulated Fertilizers on Plant Biomass of Maize

In addition to the nitrogen content in the soil, the effect of the tested fertilizers on the production of plant matter and its quality was evaluated in a pot experiment. Plant biomass production was evaluated based on the assessment of the dry weight of maize plants in terms of t_1_–t_3_ and root system size (t_3_). Biomass quality was determined by measuring chlorophyll content (N tester), development of vegetation index (NDVI), and selected photosynthetic parameters during vegetation (t_1_–t_3_).

##### Above-Ground Biomass Production and Root Size

**Figure 5:**
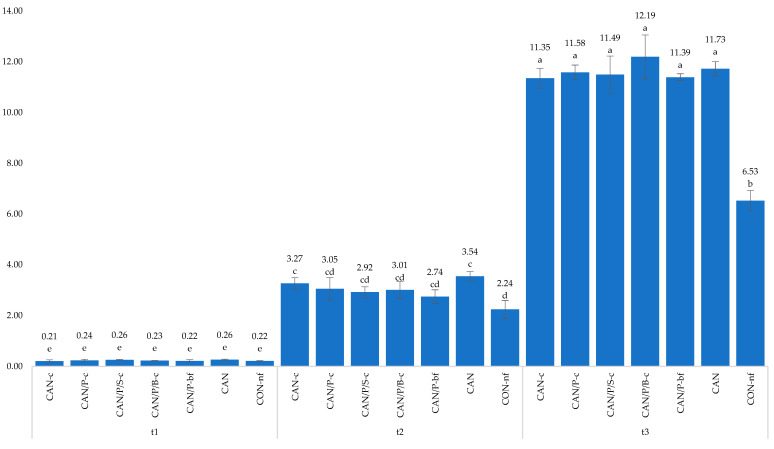
Dry matter weight of maize AGB (g/1 plant). The weights of AGB are expressed as mean (n = 4); the error bars represent the standard deviation. Different letters above error bars denote statistically significant differences among treatments using Tukey’s post hoc tests. CAN-c: 100% CAN with P3HB coating; CAN/P-c: 50% CAN + 50% P3HB with P3HB coating; CAN/P/S-c: 50% CAN + 25% P3HB + 25% struvite with P3HB coating; CAN/P/B-c: 50% CAN + 50% biomass with P3HB coating; CAN/P-bf: 50% CAN + 50% P3HB encapsulated in biodegradable film; CAN: 100% CAN (positive reference); CON-nf: without fertilizer (negative reference).

The development of dry weight of maize aboveground biomass (AGB) production is presented in Figure 5. The dry weight of AGB was not significantly affected by fertilization in the initial stage (t_1_). No significant differences between the fertilized treatments were found in other plant collection dates (t_2_ and t_3_) either. In this growth phase, the expected reduction in dry matter of AGB on the nitrogen unfertilized treatment (CON-nf) was confirmed. While the relatively highest dry matter yield of AGB was found on the CAN at t_2_, at the end of the experiment (t_3_) the highest dry matter production was obtained on the CAN/P/B-c coated fertilizer treatment.

**Figure 6:**
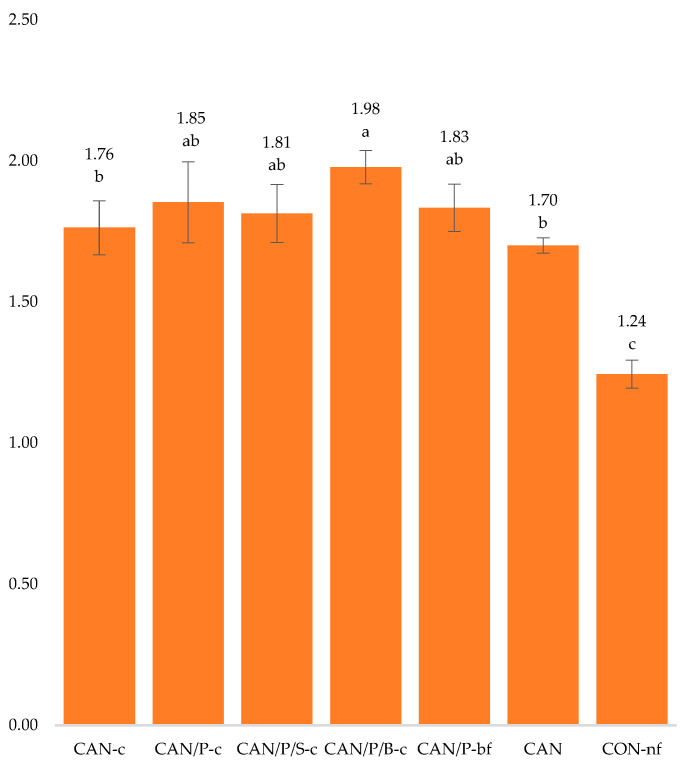
The maize root system size (nF) determined by root electrical capacitance (*C_R_*). The values of *C_R_* are expressed as mean (n = 4); the error bars represent the standard deviation. Different letters above error bars denote statistically significant differences among treatments using Tukey’s post hoc tests. CAN-c: 100% CAN with P3HB coating; CAN/P-c: 50% CAN + 50% P3HB with P3HB coating; CAN/P/S-c: 50% CAN + 25% P3HB + 25% struvite with P3HB coating; CAN/P/B-c: 50% CAN + 50% biomass with P3HB coating; CAN/P-bf: 50% CAN + 50% P3HB encapsulated in biodegradable film; CAN: 100% CAN (positive reference); CON-nf: without fertilizer (negative reference).

The size (capacity) of the root system of maize plants was determined only at term t_3_ using the so-called electric root capacity (*C_R_*). The *C_R_* values are presented in Figure 6. The size of plant roots strongly correlates with the weight of AGB dry matter in a given phase (r = 0.851; *p* ˂ 0.001). The significantly highest value was reached in treatment with relatively highest AGB production (CAN/P/B-c). On this variant, *C_R_* was 16.5% higher compared to maize grown on the variant fertilized with CAN.

##### Nitrogen Content in Plant, Chlorophyll Content, NDVI, and Quant Yield of PSII

The nitrogen content determined in AGB of maize plants decreased logically over time (t_1_ to t_3_). The effect of fertilizer application on its amount in tissues was observed mainly in treatment CAN-c (see Table 7). The nitrogen content in plants was the significantly highest in t_1_ and t_2_ after fertilization with this treatment. The nitrogen contents in the AGB of maize plants were equal in the fertilized variants at the end of the experiment (t_3_). These contents of N in plant correlate with the state of mineral N amount in the soil, especially the NO_3_^−^ form (t_1_: r = 0.729, *p* ˂ 0.001; t_2_: r = 0.753, *p* ˂ 0.001; t_3_: r = 0.845, *p* ˂ 0.001).

In addition to the N content in the AGB of maize plants, the normalized difference vegetation index (NDVI) and chlorophyll content, expressed as N-tester value, were determined (Table 7). The amount of chlorophyll in the plants was also determined using a Yara N-tester. N tester values significantly correlated with nitrogen contents in AGB (r = 0.740, *p* ˂ 0.001). Since the chlorophyll content in the plant is directly dependent on the amount of nitrogen in the tissues, this fact can be explained by the gradual release of nutrients from coated fertilizers in time and its relative deficiency in the later stages of growth (t_3_) in plants fertilized with conventional, non-coated fertilizers. While in the terms t_1_ and t_2_, the plants fertilized with uncoated CAN showed the significant highest N tester value, in the term t_3_ the significant highest content of chlorophyll was found in treatments CAN/P-c, CAN/P/S-c and CAN/P-bf.

A significant dependence was found between the measured values of N tester and NDVI (r = 0.822, *p* ˂ 0.001). However, fertilization did not have a significant effect on the values of the vegetation index, as shown in Table 7.

Quantum yield of photosystem II (*Φ_PSII_*) is a measure of photosystem II (PSII) efficiency and corresponds to the F_V_/F_M_ ratio, where F_V_ is the maximum variable chlorophyll fluorescence yield in the light-adapted state and F_M_ is the maximum chlorophyll fluorescence yield in the light-adapted state. The quantum yield thus provides an accurate estimate of photosynthetic activity. The value of *Φ_PSII_* was not significantly affected by coated fertilizer fertilization (Table 7). The actual capacity of the PSII for photochemical processes by availability of reaction centers of the photosystem II significantly corelated with NDVI values (r = 0.713, *p* ˂ 0.001). Nevertheless, its values at the end of the vegetation (*t3*) were relatively highest in plants fertilized with coated and encapsulated fertilizers (0.821), compared to the *Φ_PSI_* value on the CAN (0.808) and unfertilized treatment (0.790).

## 4. Discussion

Several controlled-release fertilizer formulations have been successfully prepared: coated pellets containing CAN fertilizer and fillers that align with the circular economy’s intentions (fully biodegradable P3HB, struvite, and biomass). The behavior and release dynamics of the fertilizer in aqueous environments and vegetation pot experiments were tested.

Boyandin et al. [23] investigated the release of ammonium nitrate in an aqueous medium in pellets with a 6-fold coating of P3HB in chloroform. Its pellets contained only 25% of ammonium nitrate, and the rest was made up of P3HB or other additives (wood flour). Testing in water in research lasted seven days. (18.3 ± 7.9)% of fertilizer was released after seven days from its coated pellets containing ammonium nitrate and P3HB, and (13.4 ± 7.9)% of fertilizer was released from its coated pellets also containing wood flour. Our coated pellets containing fertilizer with 50% P3HB, or struvite, or biomass had only 3.5–5.7% released ammonium nitrate after seven days. Pellets in foils released 11.5% of ammonium nitrate. The differences in a more extended experiment would be even more pronounced. In addition, we also achieved a small coating thickness on the pellets (approx. 12–17% of the weight of the original pellet for various formulations) due to using “green” dioxolane as a P3HB solvent [22].

The release of fertilizer into water is also described, e.g., by Rashidzadeh and Olad [47]. Their slow-released NPK fertilizer, encapsulated by superabsorbent nanocomposite and prepared via the in situ free radical polymerization of sodium alginate, acrylic acid, acrylamide, and montmorillonite in the presence of fertilizer compounds, also possessed excellent slow-release property. The release of fertilizer was 14.66% on the first day, 28.54% after one week, and 57.66% after one month.

Our coated pellets containing 50% fertilizer with 50% P3HB showed excellent resistance even after 76 days in water. After 76 days, only 20% of the ammonium nitrate was released. According to a review of controlled-release fertilizers by Lawrencia et al. [4], such fertilizer release in the water environment corresponds more to fertilizer with a synthetic polymer-based coating than fertilizer with a natural polymer-based coating. We proved the feasibility of P3HB as a filler and coating material. By adjusting filler content and coating thickness, we could design the period of the agrochemical release from the formulation. Effective P3HB coating could also be used for seed protection [48].

The pot experiment results proved coated CAN fertilizers as a possible option to improve nutrient use efficiency, reduce nitrogen losses, and minimize environmental pollution while providing nitrogen to plants more gradually during vegetation, which is also described by several authors [9,49,50,51,52]. The release of nitrogen from coated CAN fertilizers affected dynamic changes in the soil mineral N content during the vegetation of maize. Contents of N_min_ and its ionic forms (NO_3_^−^, NH_4_^+^) were determined in the soil in three terms (t_1_–t_3_). Although enough of the available nitrogen can be essential for direct plant consumption, the excessive content may inevitably increase its loss in soil [53]. One of the important aspects of coated fertilizers is the longevity of nutrient release at optimal levels for plant uptake. The application of coated CAN fertilizers showed a positive effect on the N_min_ (NO_3_^−^, NH_4_^+^) release pattern, as seen in Figures S6–S8. The statistically lowest values of nitrogen content in the soil in the t_1_ term were observed after CAN/Pbf treatment, possibly because of the encapsulation, which slowed down the hydrolysis of this fertilizer and resulted in more gradual nitrogen release. This fact possibly resulted in slower nitrification and thereby reduced loss from leaching. Positive effects were found of fertilizers coated with different types of polymers such as polyolefin [54,55], multiorganic polymer, diamide of oxalic acid [55,56], and sulfur [57] on nitrate leaching. The results obtained from t_2_ and t_3_ clearly demonstrate the effects of coated fertilizers: each treatment provided a higher amount of nitrogen (both ammonia and nitrate) in comparison with common uncoated CAN. Nitrogen after this treatment was taken up by the plants and leached in the middle and lower layers of the soil, while the coated treatments continued to provide both forms of nitrogen more gradually. One of the highest supplies of nitrogen in both later terms was provided by coated treatments CAN/P/B-c and CAN/P/S-c; a similar result was also provided by encapsulated treatment CAN/Pbf. Zheng et al. [58] similarly describe the enhanced content of soil mineral nitrogen in later vegetation stages after applying sulfur-coated fertilizers. Figure 4 describes the development of nitrogen in the soil layers over time; a similar result with gradually increasing content of N in the top layer while maintaining the high amount of nitrogen in the middle layer is also presented by Xiao et al. [59]. Their controlled-release N fertilizer coated with paper-plastic composite material (composed of paper and polyethylene) reduced nitrogen leaching and ammonia evaporation from soil.

The aboveground biomass of maize after fertilization with coated CAN treatments was, as expected, higher in comparison with unfertilized treatment and comparable with the treatment without coating (CAN). These results proved that coated fertilizers present a potential alternative to common fertilizers, as their application has no negative effect on yield while minimizing the environmental losses of nitrogen. The same conclusions are presented by Škarpa et al. [60]. Trenkel [13] even describes the possibility of lowering the application doses of coated fertilizers while achieving similar yields. Some authors also describe increases in crop yields after coated fertilizer application [61,62,63,64,65]. After the use of CRF fertilizers based on polyhydroxyalkanoate, a significant increase in total fresh plant biomass was found compared with the quick-release NPK fertilizer [66]. In our experiment, the increase in aboveground biomass was observed only in t_3_ after the treatment CAN/P/B-c in comparison with uncoated CAN. A strong correlation between aboveground biomass and root size was observed in our experiment. The highest value of root size was measured on the treatment CAN/P/B-c with the highest biomass production. The correlation between the nitrogen content (especially nitrate form) in the soil and N content in the aboveground biomass of plants was observed, the same as the correlation between nitrogen content in AGB and N-tester values. Similar findings were presented by Koning et al. [67]. The content of chlorophyll plants is dependent on the amount of nitrogen in the tissues [68]; these results can be explained by the gradual release of nitrogen from coated treatment in time and its relative deficiency in the later stages of growth (t_3_) in plants fertilized with conventional, non-coated fertilizers. Although the effect of fertilization was not significant in terms of N-tester values and NDVI index, the coated treatment CAN/P/B-c and encapsulated treatment CAN/Pbf provided one of the highest values, especially in later terms of vegetation. A single pre-planting application of these controlled-release fertilizers can fill a crop’s nutritional requirements throughout its growing season.

Our research on controlled-release fertilizers with biodegradable coating involved coated formulations with urea and P3HB filler. We were able to prepare these pellets on a quarter-operational pelletizing device and coat them in larger quantities in a coating drum. This technology could be transferable to the industry. A careful study of the biodegradation of these fertilizer formulations in soil and research into the long-term effect on soil composition under field conditions will also be needed.

## Figures and Tables

**Table 1:** Agrochemical properties of used soil in the pot experiment.

Soil Parameters	Value	Ref.
Clay	20%	[39]
Dust	27%	
Sand	53%	
Oxidizable C content (Cox)	0.80%	[40]
pH (CaCl_2_)	6.09	[41]
Cation exchange capacity	164 mmol/kg	
Noverall	0.19%	
NH_4_^+^ (K_2_SO_4_)	1.48 mg/kg	
NO_3_^−^ (K_2_SO_4_)	17.2 mg/kg	
P (Mehlich 3)	36.4 mg/kg	
K (Mehlich 3)	400 mg/kg	
Ca (Mehlich 3)	2720 mg/kg	
Mg (Mehlich 3)	214 mg/kg	

**Table 2:** Scheme of the pot experiment with maize.

Treatment	Composition of Pellets Used in Vegetation Tests	Number of Pellets (pcs/Pot)	N Dose (g/Pot)
CAN-c	100% CAN with P3HB coating	4	0.54
CAN/P-c	50% CAN + 50% P3HB with P3HB coating	8	0.54
CAN/P/S-c	50% CAN + 25% P3HB + 25% struvite with P3HB coating	8	0.54
CAN/P/B-c	50% CAN + 50% biomass with P3HB coating	8	0.54
CAN/P-bf	50% CAN + 50% P3HB encapsulated in biodegradable film	8	0.54
CAN	100% CAN (positive reference)	4	0.54
CON-c	without fertilizer (negative reference)	0	0

**Table 3:** Observed plant growth parameters.

Plant Parameter	Device Used	Terms	Ref.
Chlorophyll content (N-tester value)	Yara N-Tester chlorophyll meter (Yara International ASA, Oslo, Norway)	t1–t3	[43]
Vegetation index (NDVI)	PlanPen NDVI310 device (Photon Systems Instruments, Drásov, Czech Republic)	t1–t3	
Quantum yield of the PSII (*Φ_PSII_*)	PAR-Fluorpen FP110-LM/D device (Photon Systems Instruments, Drásov, Czech Republic)	t1–t3	[44]
Dry weight of AGB	Laboratory-scale PCB Kern (KERN & Sohn GmbH, Balingen, Germany)	t1–t3	
N content in AGB	Kjeltec 2300 device (Foss Analytical, Hillerød, Denmark)	t1–t3	[45]
Root electrical capacitance (*C_R_*)	VOLTCRAFT LCR 4080 (Conrad Electronic GmbH, Wels, Austria)	t3	[44]

**Table 4:** Average coating weights (in%) and standard deviation of the weight of the original pellets (n = 10) after applying six layers of coating.

Coating Solution	Coating Weights of the Pellets (%)
7% P3HB in chloroform with ethanol	82.4 ± 4.6
6% P3HB in dioxolane	19.9 ± 1.5
6% P3HB in chloroform with amylene	Failed to form an acceptable coating

**Table 5:** Calculated average weight (%, n = 10) and standard deviation of manually applied 6-fold coating for all pellet formulations prepared on a hydraulic press.

Formulation	6-Fold Additional Coating Applied (% of Original Pellet Weight, Average and Standard Deviation)
100% CAN	11.9 ± 1.6
50% CAN + 50% P3HB	16.9 ± 2.5
50% CAN + 25% P3HB + 25% struvite	13.8 ± 2.8
50% CAN + 50% biomass	14.4 ± 2.4

**Table 6:** The composition of six-fold manually coated fertilizer formulations prepared on a hydraulic press and tested in the aqueous medium.

Composition of 6-Fold Manually Coated Fertilizer Pellets	
CAN/P/B-_C_	50% CAN + 50% biomass + coating P3HB in dioxolane
CAN/P-_C_	50% CAN + 50% P3HB + coating P3HB in dioxolane
CAN/P/S-_C_	50% CAN + 25% P3HB + 25% struvite + coating P3HB in dioxolane
CAN-c	100% CAN + coating P3HB in dioxolane
CAN	100% CAN (positive reference)
CAN/P-_bf_	50% CAN + 50% P3HB + biodegradable polymeric film (encapsulation)

**Table 7:** Nitrogen content in the above-ground mass of maize plants (% DM), N tester value, NDVI, and quantum yield of photosystem II (*Φ_PSII_*) in maize. The values represent the mean (n = 4) ± standard deviation. Different letters denote statistically significant differences among treatments using Tukey’s post hoc tests. CAN-c: 100% CAN with P3HB coating; CAN/P-c: 50% CAN + 50% P3HB with P3HB coating; CAN/P/S-c: 50% CAN + 25% P3HB + 25% struvite with P3HB coating; CAN/P/B-c: 50% CAN + 50% biomass with P3HB coating; CAN/P-bf: 50% CAN + 50% P3HB encapsulated in biodegradable film; CAN: 100% CAN (positive reference); CON-nf: without fertilizer (negative reference).

Term of Measured	Treatments	N Content in AGB (% of DM ± SD)	N-Tester Value	NDVI	*Φ_PSII_*
t1	CAN-c	5.99 a ± 0.25	519 def ± 8	0.75 cdefgh ± 0.01	0.835 abc ± 0.006
	CAN/P-c	5.81 ab ± 0.04	529 def ± 12	0.77 abcde ± 0.01	0.835 abc ± 0.006
	CAN/P/S-c	5.70 ab ± 0.20	518 def ± 8	0.75 defgh ± 0.02	0.838 abc ± 0.005
	CAN/P/B-c	5.58 bc ± 0.08	506 ef ± 7	0.76 abcdefg ± 0.02	0.825 abcd ± 0.006
	CAN/P-bf	5.57 bc ± 0.07	506 ef ± 13	0.76 abcdefg ± 0.02	0.825 abcd ± 0.006
	CAN	5.64 ab ± 0.06	565 abc ± 7	0.76 abcdefg ± 0.02	0.825 abcd ± 0.006
	CON-nf	5.25 c ± 0.08	498 f ± 10	0.77 abcdef ± 0.01	0.833 abc ± 0.005
t2	CAN-c	3.54 d ± 0.25	574 ab ± 20	0.79 abcd ± 0.01	0.833 abc ± 0.005
	CAN/P-c	3.32 de ± 0.13	538 cd ± 9	0.80 abc ± 0.01	0.840 ab ± 0.000
	CAN/P/S-c	3.47 de ± 0.11	530 de ± 12	0.80 a ± 0.00	0.840 ab ± 0.008
	CAN/P/B-c	3.30 de ± 0.16	543 bcd ± 16	0.80 ab ± 0.01	0.835 abc ± 0.013
	CAN/P-bf	3.14 e ± 0.23	541 cd ± 9	0.79 abcd ± 0.01	0.843 a ± 0.005
	CAN	3.29 de ± 0.12	579 a ± 3	0.78 abcd ± 0.02	0.825 abcd ± 0.010
	CON-nf	1.48 f ± 0.20	397 g ± 13	0.75 bcdefg ± 0.02	0.830 abc ± 0.008
t3	CAN-c	1.57 f ± 0.04	366 hi ± 19	0.71 gh ± 0.02	0.823 abcd ± 0.010
	CAN/P-c	1.50 f ± 0.04	405 g ± 7	0.71 gh ± 0.01	0.823 abcd ± 0.010
	CAN/P/S-c	1.58 f ± 0.06	399 g ± 3	0.70 h ± 0.01	0.820 bcd ± 0.000
	CAN/P/B-c	1.55 f ± 0.06	358 hi ± 23	0.73 efgh ± 0.02	0.818 cd ± 0.010
	CAN/P-bf	1.62 f ± 0.06	388 gh ± 8	0.72 gh ± 0.01	0.823 abcd ± 0.013
	CAN	1.52 f ± 0.06	350 i ± 7	0.72 fgh ± 0.00	0.808 de ± 0.010
	CON-nf	0.80 g ± 0.04	249 j ± 8	0.62 i ± 0.05	0.790 e ± 0.008

## Data Availability

Not applicable.

## Supplementary Materials

The following supporting information can be downloaded at: https://www.mdpi.com/article/10.3390/polym14204323/s1. Figure S1: Six coating layers applied manually to pellets composed of 50% P3HB with 50% CAN: coated with a solution of (a) 7% P3HB in chloroform with ethanol, (b) 6% P3HB in dioxolane, (c) 6% P3HB in amylene; Figure S2: Manually-coated pellets of different experimental formulations (prepared on a hydraulic press); Figure S3: Encapsulation of pellets containing 50% CAN and 50% P3HB into a biodegradable film; Figure S4: The final appearance of the encapsulated pellets; Figure S5: Grown maize in Mitscherlich vegetation pots in the rain shelter; Figure S6: The ammonium (a) and nitrate (b) soil nitrogen content (mg/kg of soil) in the first term (t1) of soil collection (3 weeks after sowing/fertilization). The nitrogen contents of the different soil layers (up—upper, mi—middle, bo—bottom) are expressed as mean (n = 4); the error bars represent the standard deviation. The mean values marked with an asterisk are significantly different (*p* ≤ 0.05) from the treatment without coated (CAN) by the Tukey test (each of the soil layers was statistically evaluated separately). CAN-c: 100% CAN with P3HB coating; CAN/P-c: 50% CAN + 50% P3HB with P3HB coating; CAN/P/S-c: 50% CAN + 25% P3HB + 25% struvite with P3HB coating; CAN/P/B-c: 50% CAN + 50% biomass with P3HB coating; CAN/P-bf: 50% CAN + 50% P3HB encapsulated in biodegradable film; CAN: 100% CAN (positive reference); CON-nf: without fertilizer (negative reference); Figure S7: The ammonium a) and nitrate b) soil nitrogen content (mg/kg of soil) in the second term (t_2_) of soil collection (6 weeks after sowing/fertilization). The nitrogen contents of the different soil layers (up—upper, mi—middle, bo—bottom) are expressed as mean (n = 4); the error bars represent the standard deviation. The mean values marked with an asterisk are significantly different (*p* ≤ 0.05) from the treatment without coated (CAN) by the Tukey test (each of the soil layers was statistically evaluated separately). CAN-c: 100% CAN with P3HB coating; CAN/P-c: 50% CAN + 50% P3HB with P3HB coating; CAN/P/S-c: 50% CAN + 25% P3HB + 25% struvite with P3HB coating; CAN/P/B-c: 50% CAN + 50% biomass with P3HB coating; CAN/P-bf: 50% CAN + 50% P3HB encapsulated in biodegradable film; CAN: 100% CAN (positive reference); CON-nf: without fertilizer (negative reference); Figure S8: The ammonium (a) and nitrate (b) soil nitrogen content (mg/kg of soil) in the third term (t_3_) of soil collection (9 weeks after sowing/fertilization). The nitrogen contents of the different soil layers (up—upper, mi—middle, bo—bottom) are expressed as mean (n = 4), the error bars represent the standard deviation. The mean values marked with an asterisk are significantly different (*p* ≤ 0.05) from the treatment without coated (CAN) by the Tukey test (each of the soil layers was statistically evaluated separately). CAN-c: 100% CAN with P3HB coating; CAN/P-c: 50% CAN + 50% P3HB with P3HB coating; CAN/P/S-c: 50% CAN + 25% P3HB + 25% struvite with P3HB coating; CAN/P/B-c: 50% CAN + 50% biomass with P3HB coating; CAN/P-bf: 50% CAN + 50% P3HB encapsulated in biodegradable film; CAN: 100% CAN (positive reference); CON-nf: without fertilizer (negative reference).
