# Supplementary material for: Slow-Release Nitrogen Fertilizers with Biodegradable Poly(3-hydroxybutyrate) Coating: Their Effect on the Growth of Maize and the Dynamics of N Release in Soil
Source: Polymers (Basel). 2022 Oct 14;14(20):4323. doi: 10.3390/polym14204323 (PMC9610826; doi:10.3390/polym14204323)
Supplement: Supplementary file 1 [file polymers-14-04323-s001.zip › polymers-1957469-supplementary.pdf]

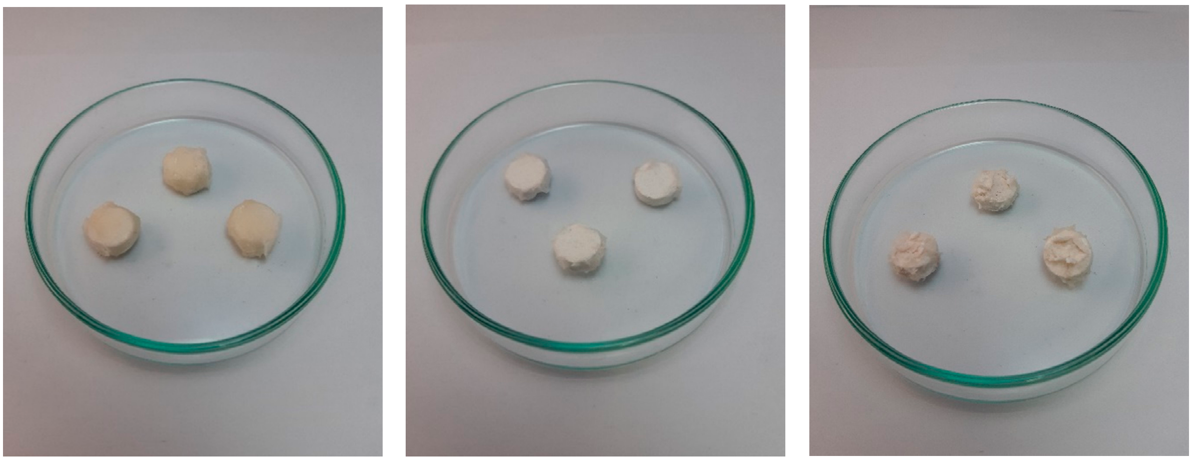

(a) (b) (c)

Figure S1. Six coating layers applied manually to pellets composed of 50% P3HB with 50% CAN: coated with a solution of (a) 7% P3HB in chloroform with ethanol, (b) 6% P3HB in dioxolane, (c) 6% P3HB in amylene.

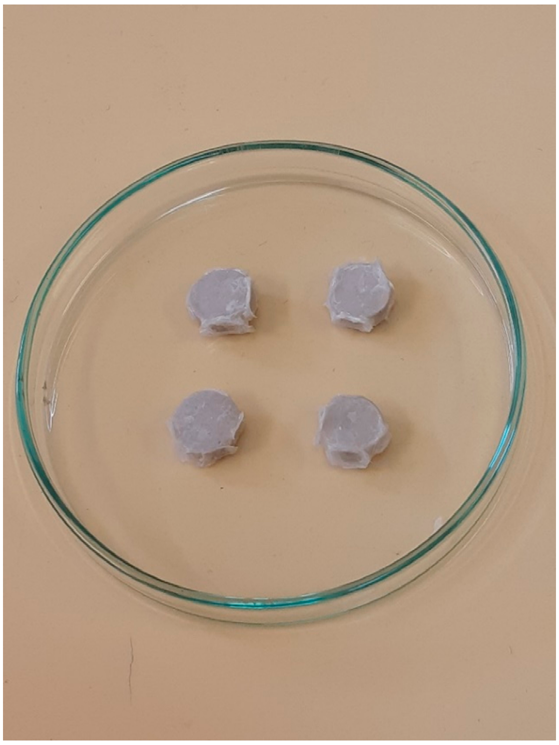

6-fold coating of P3HB in dioxolane on 100% CAN pellets

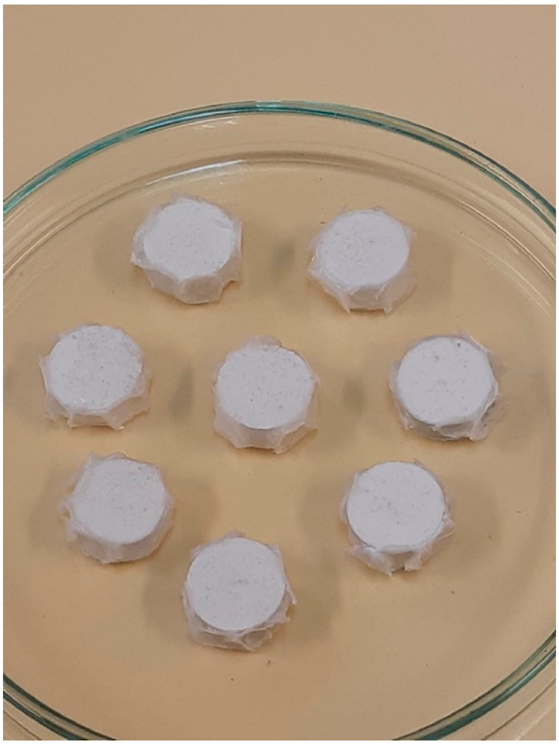

6-fold coating of P3HB in dioxolane on pellets 50% CAN + 50% P3HB

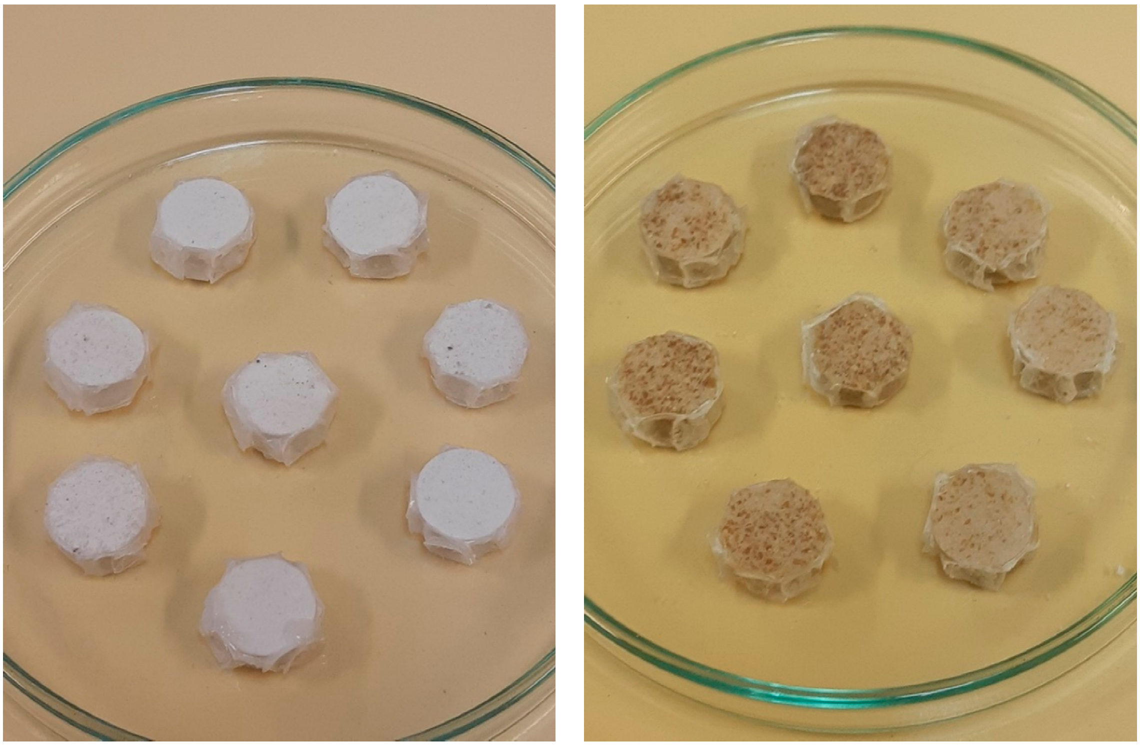

6-fold coating of P3HB in dioxolane on pellets 50% CAN + 25% P3HB + 25% struvite

6-fold coating of P3HB in dioxolane on pellets 50% CAN + 50% dried biomass

Figure S2. Manually-coated pellets of different experimental formulations (prepared on a hydraulic press).

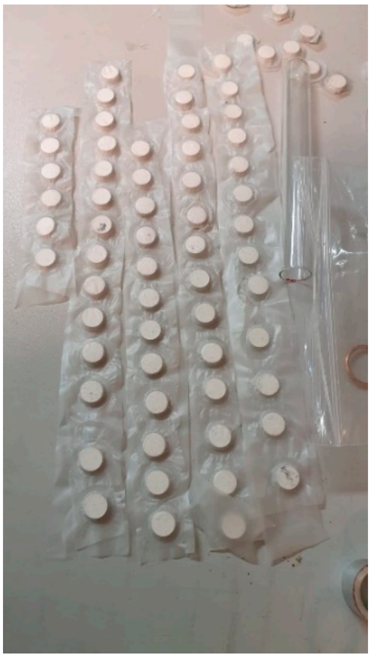

Figure S3. Encapsulation of pellets containing 50% CAN and 50% P3HB into a biodegradable film.

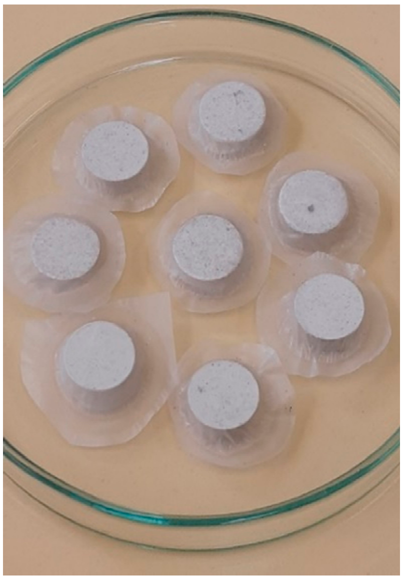

Figure S4. The final appearance of the encapsulated pellets.

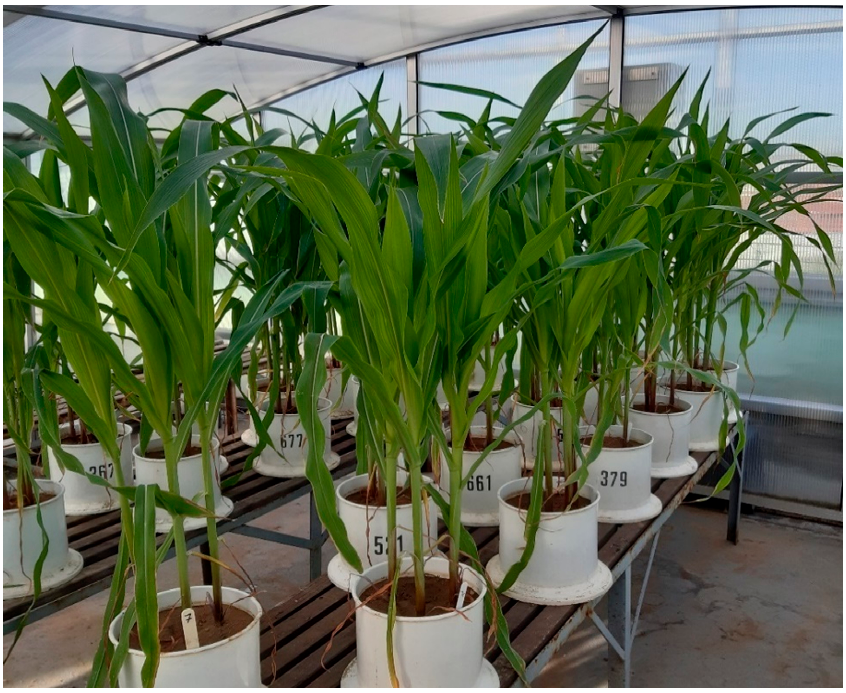

Figure S5. Grown maize in Mitscherlich vegetation pots in the rain shelter.

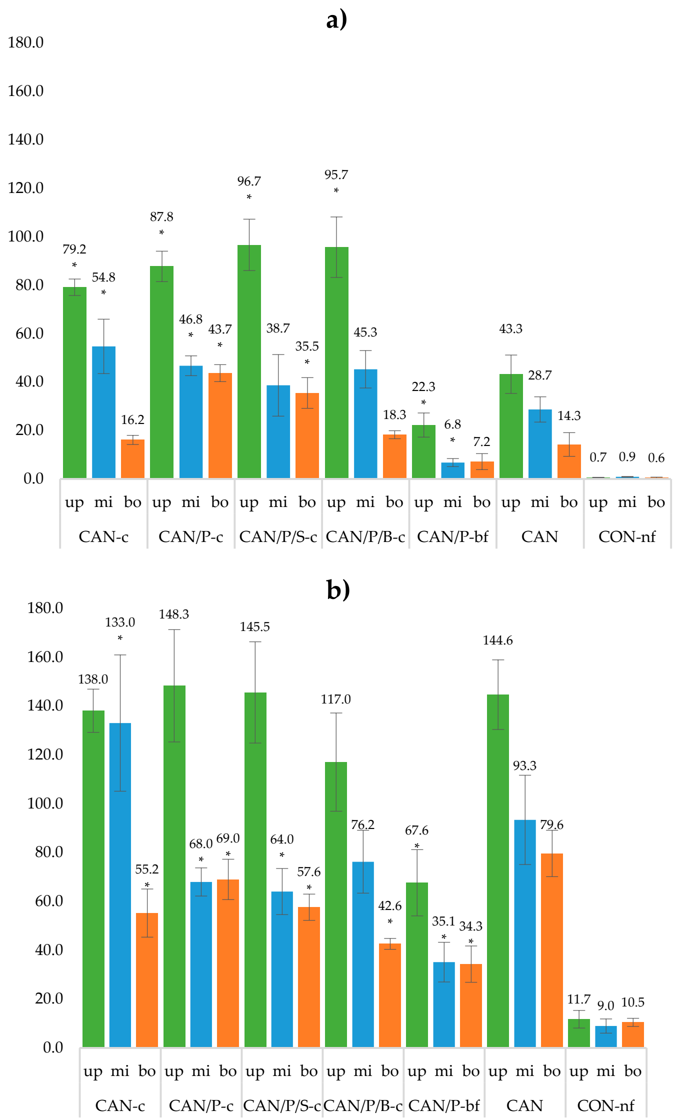

Figure S6. The ammonium (**a**) and nitrate (**b**) soil nitrogen content (mg/kg of soil) in the first term (t1) of soil collection (3 weeks after sowing/fertilization). The nitrogen contents of the different soil layers (up – upper, mi – middle, bo – bottom) are expressed as mean (n=4); the error bars represent the standard deviation. The mean values marked with an asterisk are significantly different ($p \leq 0.05$) from the treatment without coated (CAN) by the Tukey test (each of the soil layers was statistically evaluated separately). CAN-c: 100 % CAN with P3HB coating; CAN/P-c: 50 % CAN + 50 % P3HB with P3HB coating; CAN/P/S-c: 50% CAN + 25% P3HB + 25% struvite with P3HB coating; CAN/P/B-c: 50 % CAN + 50 % biomass with P3HB coating; CAN/P-bf: 50 % CAN + 50 % P3HB encapsulated in biodegradable film; CAN: 100 % CAN (positive reference); CON-nf: without fertilizer (negative reference).

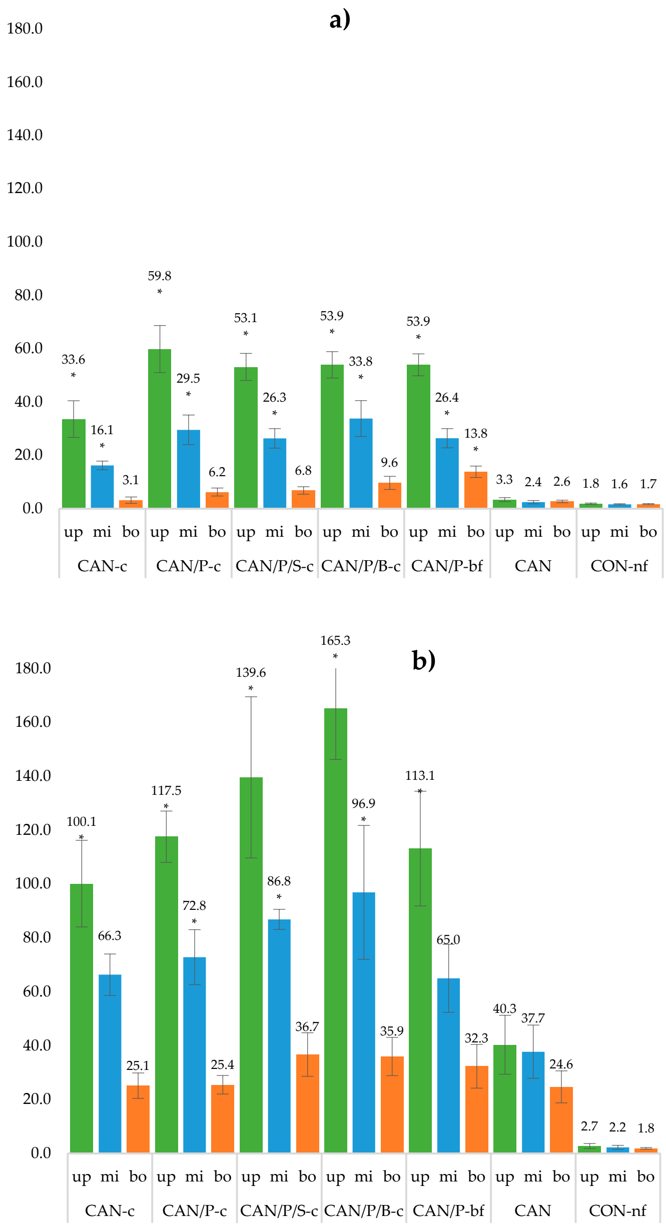

Figure S7. The ammonium (**a**) and nitrate (**b**) soil nitrogen content (mg/kg of soil) in the second term (t_2) of soil collection (6 weeks after sowing/fertilization). The nitrogen contents of the different soil layers (up – upper, mi – middle, bo – bottom) are expressed as mean (n=4); the error bars represent the standard deviation. The mean values marked with an asterisk are significantly different ($p \leq 0.05$) from the treatment without coated (CAN) by the Tukey test (each of the soil layers was statistically evaluated separately). CAN-c: 100 % CAN with P3HB coating; CAN/P-c: 50 % CAN + 50 % P3HB with P3HB coating; CAN/P/S-c: 50% CAN + 25% P3HB + 25% struvite with P3HB coating; CAN/P/B-c: 50 % CAN + 50 % biomass with P3HB coating; CAN/P-bf: 50 % CAN + 50 % P3HB encapsulated in biodegradable film; CAN: 100 % CAN (positive reference); CON-nf: without fertilizer (negative reference).

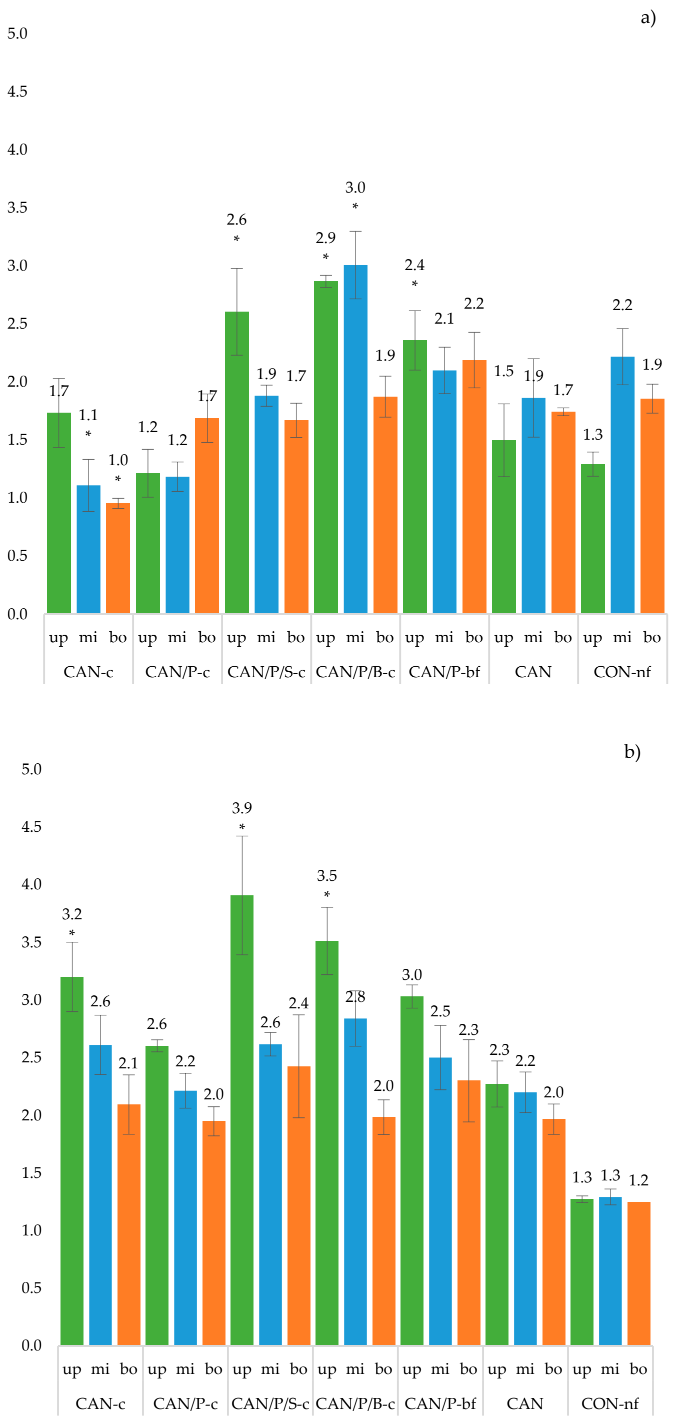

Figure S8. The ammonium (**a**) and nitrate (**b**) soil nitrogen content (mg/kg of soil) in the third term (t_3) of soil collection (9 weeks after sowing/fertilization). The nitrogen contents of the different soil layers (up – upper, mi – middle, bo – bottom) are expressed as mean (n=4), the error bars represent the standard deviation. The mean values marked with an asterisk are significantly different ($p \leq 0.05$) from the treatment without coated (CAN) by the Tukey test (each of the soil layers was statistically evaluated separately). CAN-c: 100 % CAN with P3HB coating; CAN/P-c: 50 % CAN + 50 % P3HB with P3HB coating; CAN/P/S-c: 50% CAN + 25% P3HB + 25% struvite with P3HB coating; CAN/P/B-c: 50 % CAN + 50 % biomass with P3HB coating; CAN/P-bf: 50 % CAN + 50 % P3HB encapsulated in biodegradable film; CAN: 100 % CAN (positive reference); CON-nf: without fertilizer (negative reference).
